# Blank canvas or under construction? Examining the pre-academy experiences of young developing professional team sports athletes

**DOI:** 10.3389/fspor.2023.990617

**Published:** 2023-03-15

**Authors:** Foivos Papastaikoudis, Rosie Collins, Dave Collins

**Affiliations:** ^1^ Department of Sport, Health Sciences and Social Work, Oxford Brookes University, Oxford, United Kingdom; ^2^ Grey Matters Performance Ltd., London, United Kingdom; ^3^ Moray Hopuse School of Education and Sport, University of Edinburgh, Edinburgh, United Kingdom

**Keywords:** lose talent development, coping skills, challenge, post-traumatic growth, talent pathway, social support

## Abstract

**Introduction:**

Extensive research has been carried out on Talent Development (TD) environments and an increasing amount of work shows the development of psychological characteristics as an important formal part of the academy experience. Importantly, however, very little attention has been paid to what types of skills, if any, young players arrive with. In other words, there seems to be an assumption that young athletes arrive at the academy as a blank canvas.

**Methods:**

As such, to investigate whether players' arrive with these psychological characteristics, we looked across a sample of young footballers' and rugby players' personal experiences (such as, family input, sporting background or personal challenges) prior joining the academy. Individual semi-structured interviews were conducted, and data were analysed via thematic analysis.

**Results:**

Our findings suggested that young athletes acquired an aptitude from general experiences, whereby they had already started to develop and deploy specific skills (such as, reflective practice, mental skills or seeking social support) to navigate particular challenges, prior to arriving at the academy.

**Conclusion:**

Implications include the need for coaches and psychologists to assess young athletes' skillsets and pre-academy experiences upon arrival and use this as a starting point for developing tailored and individualised pathways that would enable them maximise their potential.

## Introduction

A wealth of recent findings has suggested that young athletes follow an individualised, complex and, ultimately, non-linear trajectory to the top (1, 1–4). Given that a talented individual can be viewed as someone who possesses the potential to perform at a high level, research has focused on the skills required to negotiate the challenging pathway and realise this potential (5). Notably, a growing body of research has alluded to the central role of psychological characteristics in facilitating the development of talent (7, 6–9). Resultantly, current applied literature has focused on providing clear guidelines through which to ensure young athletes develop, deploy, and refine a toolbox of psychological skills along the TD pathway [e.g., (10–12)]. However, the extent to which young athletes already have (albeit early versions of) psychological skills when entering the academy system remains largely undiscovered. This knowledge gap should be of particular interest given the important role better refined coping skills through the pathway can play in an athletes' ability to be successful (13).

Whilst most of the research solely focuses on the full TD pathway [e.g., (4)], Williams and MacNamara (14) recently attempted to bridge some of the existing gaps in literature by examining the experiences of young athletes in the early years of the pathway. Supporting previous findings in the area (4, 15–17), results indicated that young athletes utilised a range of psychological skills (e.g., commitment, goal-setting and performance evaluations, self-belief) and diverse social support to deal with developmental challenges.

Although some key assumptions could be made regarding the *types* of skills that young athletes utilise in the early years of the pathway, it is still unclear whether these skills were developed from these early academy experiences, or pre-academy entry. Therefore, another important point of exploration concerns the precise nature of *how* these characteristics are acquired. For example, there is increasing agreement that challenge plays a big role in developing these aforementioned skills [e.g., adversity related growth, (3, 18)]. Simply put, research suggests that adversity-related experiences are integral to testing and refining skills, as well as developing attitudes that performers already possess (4, 19). Notably, however, research has solely focused on the experiences of young athletes on the TD pathway (4, 20). Given the much less structured pre-academy pathways, it seems unlikely that young athletes acquire these important skills in such a formal manner through that period, indicating that similar development may also occur in unstructured ways [cf. comparisons between formal and unstructured development of musicians – (21)]. In short, development may be facilitated prior to joining the more formal academy systems but exactly how is unknown.

Moreover, the selection of young performers into highly structured TD pathways now occurs at an increasingly younger age (22). In an effort to identify and recruit the most talented young players, many football academies in the UK require players as young as six to attend several weekly training sessions, before their formal registration begins at 8 years of age (23, 24). As such, it could be argued that the exposure to a range of challenges prior to formal entry seems to be inevitable. Therefore, the early experiences of young athletes at a pre-academy level in preparation for the challenging and, often, unpredictable academy pathway should now become an area of great interest.

Reflecting the above, another important question extending beyond the timing of this development, concerns the mechanisms that underpin growth before entry. Namely, to clarify whether psychological skills are present before the occurrence of challenge (as experienced within the academy pathway) or if challenges themselves provide a platform for these skills to be developed. Against this distinction, previous research has suggested that the experience of negotiating challenges can generate growth through the process of deploying existing psychological skills (14, 20, 25, 26). Whilst the nature and incidence of learning-specific and general challenge before entry remains largely undiscovered, this debate is of even greater importance.

To summarise, several TD studies in sport have indicated that more successful athletes made use of better refined coping skills through the pathway [e.g., (13, 27, 28)], but when and how they learned these skills remains unclear, prior to their exposure to the formal tuition and testing regimes which characterise effective academies. Therefore, the aim of the present study was to examine the extent to which these young players arrived with skills or as a blank canvas when entering an academy. Specifically, first of all, we explored the types and origins of coping skills these young players already had developed prior to entry, and secondly, the *role* of challenge in the learning and development of these skills. Indeed, if challenge played a role, then we looked to explore the perceived nature and timing of these challenges for the young football and rugby players who had just entered the academy system.

## Methods

### Research philosophy and design

Aiming to find solutions to real world problems through the initiation of practical knowledge, a pragmatic research philosophy (29) was deemed most appropriate in this study. Unlike other paradigms whose processes are largely driven by matters of ontology and epistemology, pragmatic research is primarily interested in answering questions that are important for those in applied settings (30). Nevertheless, it is important to highlight that pragmatic enquiry is undertaken from a clear philosophical base (31) which guides all stages of the research process. Thus, instead of focusing on the elicitation of generalised realities or subjective constructions, our efforts were channelled into the identification of applied artifacts that would deliver applied impact for coaches, practitioners and key stakeholders (29) in the context of TD in sport. To explore the skills, experiences and reflections of young athletes who have recently arrived at formalised TD pathways in sport, a qualitative methodology was adopted (32). Importantly, we also considered ourselves, the researchers, as co-constructors of knowledge (33). Therefore, in accordance with the basic tenets of pragmatic philosophy, this study was facilitated by our applied knowledge, skills, and experiences of working and performing in elite sport talent pathways (29). In this regard, Bryant (34) posited that, practical knowledge and personal biases can offer an innovative insight. In essence, research-derived knowledge and experience-derived knowledge (34) both feed into each other as applied settings evolve (35).

### Participants

Given the stated aims of this study, we set out to recruit young performers from four elite academies in two professional team sports. Specifically, 14 young male performers (seven football players and seven rugby players) aged between 10 and 14 years were purposively recruited. We requested clubs to nominate players they identified as having the most potential to achieve at the highest level. Their recent arrival at academy systems was intended to support accurate recall of events experienced prior to entering an academy system and address issues that might have arisen in retrospective interviews with older performers. More specifically, varying participant ages occurred due to sport specific differences, whereby football players can enter the academy system at the age of 8 while rugby players do so at the age of 13. In short, all were within two seasons of academy entry.

### Procedure

Once ethical approval was obtained from the authors' institutional ethics committee, professional sport academies were initially approached and provided with a written proposal about the purpose, the procedures, the timescale (and input required from participants), and planned outcomes of the study. Participants meeting the criteria were invited to participate through personal contact, *via* a gatekeeper (for example, the age-group coaches), then informed consent was gained from each participant prior to their interview with confidentiality assured. As players were all under the age of 18, parental consent, along with participant assent, was also secured.

To facilitate the ease of discussion and ensure consistency throughout the interviews, a semi-structured interview guide was developed (see Table 1). The guide consisted of open-ended questions and relevant follow-up prompts and probes adjusted to suit the background of participants (e.g., age, stage of development) with the aim of gleaning as much pertinent information as possible. Specific probes and prompts were also used for clarification and elaboration of key points and to obtain consistency in the depth of responses (36). This guide was informed by relevant TD literature and conducted on the base of a retrospective-tracking protocol that has been previously employed to draw out speciﬁc details on personal experience (37, 38).

**Table 1:** Interview guide.

Purpose	Question	Prompt	Analysis
Examination of Pathway prior to joining the academy; key incidents, identified critical incidents.	Using a timeline, can you draw me your pathway up to joining the academy?	When did you start and how? What sports did you take up? What did your early experiences look like? What did they mean to you? What were the biggest ups and downs (i.e. sport and/or life challenges)? What were the biggest learning experiences? What did you learn that was subsequently useful? For example, specific skills?? *Using the timeline drawn by participant:* What helped you the most here? *(Pointing to challenging occasions)* Who helped you the most here? *(Pointing to challenging occasions)*	Nature of Involvement Measure and description of early experiences Measure and description of past critical incidents Major critical incidents – stand out as being significant incidents Positive and negative developmental impact of challenges Psychological characteristics possessed, developed, deployed What skills did they have? Skills developed through challenges. Social Support
Retrospective examination of specific critical incidences	What were the most difficult experiences or events (if any) you had prior to coming into the academy?	Why was it challenging? Were you prepared for it? What helped you the most? How? Who helped you? How? What do you think you learned from it? Can you give me examples? What would you have done differently?	Major critical incidents Positive and negative developmental impact of challenges Did they possess the skills to make the most of the opportunities available? Skills developed through challenges. Social Support

Data collection was arranged in two parts. In an effort to ensure accuracy and validity of recall, participants were given the opportunity to anchor their recall of incidents to particular times and events. According to Drasch and Matthes (39), this approach can address some of the limitations of retrospective recall inherent in this method of data collection by ensuring participants related their experiences to the key stages that applied to their own pathway. Therefore, in the first stage, participants in collaboration with the interviewer developed a trajectory chart of their career on a standardised grid. Following this, guided questioning was implemented utilising the standardised interview guide. This allowed for an in-depth examination of the different experiences encountered along the TD pathway encompassing both sport and non-sport related events. Building on this stage of questioning, the second part addressed a retrospective reflection on “traumatic” or impactful events including psychological challenges experienced, methods employed, significant other/coach inputs and lessons learnt.

### Data analysis

All interviews were transcribed verbatim with each interview lasting approximately 30 min (20–40 min). Following this, and using the self-drawn trajectory charts, participants' experiences were tracked across the pathway process. Drawing on these retrospective accounts, alongside players' subsequent viewpoints, inductive content analyses were conducted (40). This encompassed reading and re-reading the transcriptions followed by employment of qualitative analysis software (QSR NVIVO 9) to transform raw data units into thematic hierarchies by engaging in tag creation, category creation, and category organisation (41).

### Trustworthiness

All interviews were conducted by the first author who developed trust with the participants, achieved by demonstrating a genuine appreciation of their history and current situation, as well as the demands of their development and performance experiences. Essentially, this process was further facilitated by the authors' roles in TD pathways and as such, knowledge of the themes being discussed.

Trustworthiness of the data analysis process was also facilitated by QSR NVIVO's optimisation of transparency [cf. (42)]. A reflective journal was maintained by the lead researcher to reduce the likelihood that interpretative bias affected the data analysis (36). The use of constant comparison method provided the platform for challenging first author's data interpretations (35). Specifically, the second author read the full transcripts of all 14 interviews and reviewed the labels/codes created by the first author. When discrepancies in interpretations were found and/or concerns about potential interpretative bias arose, reflective discussions took place until a mutual consensus between authors was reached (43). To further ensure that the first and second authors remained mindful of their assumptions and presumptions, the third author acted as a “critical friend” throughout by generating rigorous scrutiny and in-depth exploration of the interpretations, explanations and meanings emerged from the data analysis process (44).

Additionally, analysed interview transcripts were returned to each participant *via* email to allow subsequent member reflections (45). This process involved a 10–20-min face-to-face conversation to discuss the emerging results, specifically the accuracy and validity of quotes considered for inclusion in the paper from that individual together with any additional reflections which occurred to them. Indeed, eight participants provided additional details regarding their experiences and skills developed pre-entry, further enhancing the robustness and richness of our findings. Pertinent information was reintegrated into the process of data analysis. Finally, feedback was sought on the researcher's interpretation of these quotes and the context of the results subsection in which they would appear.

## Results

The aims of this investigation were to explore (1) the types/sources of coping skills these young players already had or have developed to deal with early challenges and how these evolved, and (2) the nature and timing of challenges perceived to have helped young football and rugby players who had just entered the academy system to learn and develop their coping skills *before entry*. The results begin with an overview of the participants' self-reported coping skills, as displayed in Table 2. For clarity, these results were developed from the process of reflecting on their pre-academy experiences, including the process of navigating and negotiating challenges. Secondly, we then consider participants' experiences through which these coping skills were acquired. These themes are displayed in Tables 3, 4 in a chronological order. All these themes are presented in the results section with exemplar quotations to illustrate the analysis and the percentages of participant reporting each theme. These percentages are displayed to demonstrate the frequency with which participants offered certain responses, they are not intended to display any differing importance or value of the findings.

**Table 2:** Self-reported coping skills *.*

Umbrella Theme	Higher Order Theme	Lower Order Theme	Exemplar Quotes
Mental skills 100%	Motivation 100%	Genuine love for the sport 86%	“I used to see it as a challenge. But I was not really worried about it. It's more about playing and enjoying the game. When you’re younger, all you want is to have fun, so I let myself enjoy it” (F2)
		Desire to develop and succeed 86%	“I was a lot more determined than other players. You know… a personal drive… I did a lot more extra training than the other players. I wanted to improve, I wanted to learn the game and get to their level” (R4)
		Willingness to put in hard work 93%	“It was a rocky start, a bad couple of sessions I remember. But that motivated me to work harder in and out of training” (F6)
		Motivation to prove others wrong 21%	“I wanted to prove them wrong, that they made the wrong decision. I wanted to show them what I was capable of. If you are being passionate about something, you will achieve it.” (R6)
		Motivation to make people proud 50%	“Because my Granddad always wanted to come and watch but couldn’t. So, when he died, it motivated me more to try harder and get to the top.” (F5)
	Self-efficacy 100%	Belief could excel despite challenges 86%	“Well, I was playing against some of the best players in the area. I used not to think about it when on the pitch… I was confident in myself; I knew I could deal with it.” (F1)
		Confidence to engage with challenges 86%	“I loved the idea of playing against the best and being watched by the best coaches in the area. I mean, it makes it more exciting” (R2)
		Setting high personal standards 67%	“I discussed this with my dad. We decided that signing for Academy B would make me a better player, it was more challenging. You need to be confident to push yourself out of you comfort zone” (F5)
		Unshakable self-belief despite other's doubts 21%	“Even though most of my friends were doubting my skills, I knew I had it… I believed in myself, I believed that I could become the best.” (R4)
	Focus and distraction control 71%	Ability to focus on the task at hand 50%	“I did not want to let my emotions get the better of me, I focused on what I was about to do and dealt with mistakes after” (F3)
		Focusing on own goals 50%	“My goal was to progress and be the best I could be. It was hard leaving friends behind but had to think long term” (F5)
		Ability to block out distractions 43%	“I did not want to let their opinion distract me so I just tried not to listen to it. I focused on the feedback from my coaches and worked hard on it.” (R4)
		Using distractions as a coping mechanism 21%	“So, I tried to have fun, like playing XBOX instead of focusing on my injury all the time. Doing stuff that you can without putting stress in your engine.” (R2)
	Self-awareness 64%	Awareness of strengths and weaknesses 64%	“You must work at it all the time. You have to focus on the professionalism. Most of my friends were talented and I wasn’t talented at all, but I was passionate. My attitude was my biggest strength.” (R4)
Soliciting and using social support 100%	Identifying available social support 100%	Recognising the value of social support 100%	“It was hard (i.e., injury) and my parents were always there for me, whatever I needed. They were saying positive things like ‘it is going to be fine’ and we would do things that would make me feel better” (R2)
		Distinguishing between positive and negative social support 50%	“Unlike my teammates, my coach was very supportive. I was disappointed, but he showed confidence in me. He said, ‘if you take my feedback on board and work hard, you will be in the first team squad soon” (R4)
	Mobilising social support resources 100%	Soliciting and using social support when needed 100%	“I was friends with N and E from grassroots, so they helped me a lot. They would speak to me in the dressing room and introduce me to the others. They made me feel comfortable.” (F2)
		Managing the impact of social support 50%	“There was a lot of pressure, they believed in me. But, telling me ‘You have the attributes, you need to be the best etc’ didn’t work for me. So, I decided to talk to them and explain how I felt.” (R3)
Learning skills 100%	Reflective practice 100%	Ability to reflect and make sense of own experience 86%	“I tried to dribble past them but failed. They were older, stronger, and taller than me. I wouldn’t be able to match them physically. I realised that I needed to change my game, move the ball quicker” (F4)
		Identifying areas for improvement 64%	“I discussed this with my dad and decided that I needed to improve my physicality, to become faster and stronger” (F6)
	Identifying and using learning from previous experience 100%	Perceiving challenges as growth opportunities 86%	My coach was saying ‘you either win or learn’. Nobody wants to struggle with things, neither do I. But those struggles are opportunities to learn.” (R7)
		Identifying and applying lessons learnt 71%	“I learnt how to focus on getting the next thing right and not thinking about a previous mistake as this would make me angry” (F7)
		Draw confidence from prior experience 86%	“During trials I had to play against older players but didn’t affect me. In grassroots, I had played against older boys a couple of times, so I had the confidence to do it again.” (F2)
	Identifying and using learning from others’ experiences 86%	Ability to learn from others’ experience 71%	“The way my dad handled it taught me how to stay strong and positive whatever the situation is. He was there for my mum who was having chemotherapy, he supported me and my brother, always being positive and optimistic. There was not a moment making us feel something would go wrong.” (R1)
		Applying learning from vicarious experience 64%	“My brother was playing for the older group and obviously prepared me a lot. I used to watch him training so I knew what to expect and what I needed to do” (R2)

**Table 3:** Nature of general experiences as part of early involvement with sport*s.*

Umbrella Theme	Higher Order Theme	Lower Order Theme	Exemplar Quotes
Initiation to sports 100%	General Experiences 100%	Early exposure 100%	“I started playing rugby when I was 3. I was quite young really” (R1)
		Fun and challenging activities 100%	“When he used to come around, he’d bring a football and some cones, and he’d teach me how to dribble in and out of the cones.” (F3)
		Multi-sport experience 100%	“I have done loads of different sports in my life. For example, I used to play football. I have also played some basketball which I really enjoyed as part of PE at school.” (R6)
		Early success 86%	“I was playing for a local football club and after a few months, the coach told my dad I was getting on really well and maybe they should take me to a Sunday League Club. I was so happy” (F6)
		Playing for a team 100%	“I started playing football when I was about four. I was young, playing for a local team.” (F2)
		Watching sporting events 100%	“I was watching sports since I was like 3 or 4. It is part of the family, we were going to the matches and supporting the local club.” (R7)
		Observing family members 64%	“My dad was coaching a rugby team at the time and would get me along to watch his training sessions. It was part of the family culture and quickly passed on to me” (R2)

**Table 4:** Nature of reported challenges Pre-entr*y.*

Umbrella Theme	Higher Order Theme	Lower Order Theme	Exemplar Quotes
Challenges 100%	Sport 100%	Academy trials 64%	“My first training session on trial was tough, much harder than the Sunday League. I didn’t think it was going to be that hard. It took me some time to settle. It was very competitive; everyone's eyes are on you” (F6)
		Playing up 50%	“I remember when I was at the Development Centre, they got us to train with the U9s a couple of times. It was a very hard challenge” (F7)
		Underperformance 43%	“I would fume inside; I couldn’t afford making mistakes. I needed some time to get my head back up after poor mistakes. At the time, I thought of a bad performance as a step back. It could make me sad” (F3)
		Physical 36%	“I think I could keep up with the technical side, it was mainly the physical side. Whenever you tried to get on the ball, they would do it faster than you. They were stronger and better in reading the game.” (F5)
		Game changes 36%	“Obviously being used to tag rugby moving on to touch rugby was a bit of challenge. I needed some time to fully understand the game and tactics. It requires different mentality and approach” (R6)
		Rejection 21%	“I was in the third team and I was quite upset by that because I could see the other players in the first team, they were starting for the first team all the time and I was a bit jealous” (R4)
		Late start 21%	“It was the lack of experience, I struggled to understand the game, where to go, how to move. I was really enjoying the game but could not follow the pace. There's much to learn while others had already progressed” (R3)
		Injury 14%	“It was big to hear that I would be out of sports for the whole summer… Yeah that was hard because when you are young all you want is you know… running and playing around and that is it” (R1)
		Choosing between academies 14%	“When I got 8, I had to sign. I was at Academy A and Academy B at the same time, but I had to decide. It was very difficult because they’re completely different teams. I also had very good friends” (F5)
	Environmental/Social 64%	Adapting to new environments 43%	“When I was young, going into the dressing room at seven, I cried. It was scary. Because I was so young, I didn’t know many people here, so it was all different people, it was nerve racking coming in.” (F2)
		Criticism from significant others 43%	“During trials, my friends were in the first team and I was in the second. They were like ‘how come you’re doing this, you shouldn’t be going to do it’. That was initially dragging me down a lot.” (R4)
		Leaving family/ friends behind 36%	“One of the downs was probably picking a team, between Club A and Club B because it was leaving people behind. But also, I knew either of the decisions were good. Leaving my friends behind was tough though.” (F5)
		Pressure from significant others 21%	“When I started playing rugby, there's a lot of pressure on me. Because of my size, people expected me to be dominating the game. Well now I am that player… but back then I was not aware of the rules, I did not know the game… I was lost.” (R3)
	Family 29%	Bereavement 21%	“I lost some family members and that was traumatic really. It's always a shock… I was shocked. I had to deal with the idea of them not being around anymore” (F4)
		Illness 7%	“My mum was diagnosed with cancer in 2013. That was probably the most difficult challenge I have ever been through. A huge down… I could not focus on my rugby and school. But, how could you really?” (R2)
	Logistical 29%	Balancing commitments 29%	“At the time, I found it difficult to balance commitments. I was playing school rugby with the 11s and 12s, traying with a club once a week plus some swimming. It was tiring really” (R5)
		Travel commitments 7%	“Before I started playing rugby, I wanted to join a football club. But my mum couldn’t support me as we lived far away from the training centre. That was a big down as I had many friends playing for that team” (R3)
	Educational 7%	Drops in school performance 7%	“Then in year eight, I started getting distracted from school because I was so worried about my rugby. I realised that I was not getting on well and I had to balance it” (R4)

### Coping skills

When exploring the skills developed and applied pre- academy, all participants were able to note a number of coping mechanisms. Specifically, these mechanisms were conceptualised as mental skills, soliciting and using social support, and learning skills (see Table 2).

#### Mental skills

Support for the development and application of mental skills before entry was pervasive throughout the data. As shown in the Table 2, participants commonly referred to motivation as an important mechanism for coping with early memorable challenges. Among others, they reported utilising motivation to handle different challenges including the stressful period of academy trials, criticism from others and non-sporting incidents such as loss of a family member. Essentially, motivation was described as being demonstrated in a variety of ways, including genuine love for the sport, desire to develop and succeed, willingness to put in hard work, motivation to prove others wrong and making people proud*.* It was also recognised more generally in terms of overall motivation. For example, R5 described, “motivation is a key factor determining your success in whatever you do, whatever you’re going through… No matter how difficult a challenge can be, it's my motivation that drives me.”

In addition, self-efficacy was also consistently identified as another key mechanism in facilitating the participants' endeavours in the early years. Once again, the way self-efficacy manifested varied among participants, with analysis revealing several sub-themes such as belief to excel despite challenges, confidence to engage with challenges, setting high personal standards and unshakable belief despite others' doubts. Importantly, both the motivation and self-efficacy that propelled the participants' efforts in handling these situations seemed to be present *before* the occurrence of recalled incidents.

Moreover, in their accounts of events the participants highlighted focus as a key mental skill for effectively negotiating early challenges. This focus was predominantly directed to specific goals that took either the form of process targets that were within the participant's control (e.g., making the right decisions) or outcome targets (e.g., be successful on trials). The importance of distraction control was also discussed by participants. Specifically, they referred to an ability to *block out* distractions and direct attention to the most important aspects of development and/or performance. Interestingly, however, some reported *using* distractions as a means of dealing with difficult challenges (e.g., long-term injury).

As another key mental skill, participants noted how *self-awareness* of their strengths and weaknesses helped them to handle early challenges. Importantly, this heightened awareness and, more generally, the process of skill-refinement was often facilitated by social support factors and underpinned by an ability to reflect on personal experiences (i.e., learning factors), both of which mechanisms are discussed next.

#### Soliciting and using social support

All participants discussed the value of identifying and mobilising social support in navigating their way through the developmental “ups and downs”. In this context, family support played a key role. Among others, this involved parents providing emotional support after setbacks, offering feedback, sacrificing resources to offer extra training and older siblings encouraging the participant prior to and during the intense period of academy trials. Albeit less discussed within the interviews, coaches and friends seemed to also form another part of the participants' support network. Notably, coaches often acted as a source of self-efficacy by displaying genuine appreciation of the participant's circumstances whilst providing specific feedback tailored to support their needs. Interestingly, participants indicated that social support was not merely utilised in a reactive manner to negotiate challenges but also proactively in preparation for future challenges.

Although the participants often indicated a preference for utilising one support resource over another, the strength of coherence among one's support networks was also outlined. The following excerpt of a rugby participant whose mother was diagnosed with cancer epitomises how a coherent support network can be key to effectively navigating a memorable life challenge:

It was a big trauma. My dad and my older brother were both really supportive. I think these moments bring families closer. My dad would give us courage… He said positive things like ‘Everything is gonna be fine, we need to be patient’. My older brother provided emotional support, he listened to me. My friends and teachers at school also offered to help. My friends know me … I am a bit close as a person but being there for me was reassuring. Eventually, I used their support, and this was a bit of a relief (R1).

Despite the apparent advantages of such coherent networks, this was not always viable as not all support environments were identified to be positive by the participants (e.g., friends being a negative influence). Thus, employment was underpinned by an ability to distinguish between positive and negative support environments. In this regard, participants commonly stressed the need for careful and effective *management* of social support resources available. Essentially, the participants asserted that social support should be tailored and deployed in an appropriate way.

#### Learning skills

Another crucial attribute consistently reported by all participants was learning skills. The ability to make sense of experiences and outline areas for improvement through the use of reflective practice was reported by all participants. Interestingly, participants commonly alluded to the value of reflective practice both from micro-level and macro-level perspectives. This process was portrayed as an in-depth evaluation of an experience that enables the discovery of underlying reasons of a challenge. This is best illustrated by the quote below:

“It was very different from Sunday League. I had to catch up. They were physically better, they also made quicker decisions. Me and my dad sat down and discussed what and how to improve. We thought I needed to work around my fitness, trying to get stronger and faster. This was something that would make me a better player” (F6)

Of course, as implied here, there appears to be clear aspects of learning resulted from this reactive process. Such learning was reportedly implemented by athletes in a proactive manner to reduce the likelihood of similar challenge occurring in the future. This signified an ability to identify and apply learning from previous experiences of handling developmental challenges.

Interestingly, vicarious experience was identified as another source of learning experience that helped performers cope with memorable challenges. To clarify, participants reported drawing knowledge, experience and, ultimately, confidence in an ability to cope with challenges from observing and reflecting on significant others' experience.

### Nature of experiences

Tables 3, 4 depict the nature of the experiences before entry, and the frequency with which they were reported. Experiences are presented in chronological order to provide an insight into the process that facilitated the genesis and subsequent development of coping mechanisms shown in Table 2. Data indicate that, as a result of their general experiences participants acquired a tendency or aptitude which then became synthesised into a more focused skill as a result of a challenge. Indeed, all general experiences reported occurred between the age of 2–7 with first memorable challenges reported no earlier than the age of 6 (*M* = 7.8_years_, SD = 1.2). Importantly, further evidence of the genesis of these skills was shown in Table 2. Social support and learning factors formed part of the broader upbringing along with the general experiences reported to have played an important role in the early development of this identified aptitude.

#### General experiences

Participants were asked to reflect on their initiation and start in sport. All participants reported an early exposure in sports between 2 years old and 7 years old (*M* = 3.8_years_, SD = 1.1). The family influence was referenced by many as an inspiration to take up sports. Indeed, participants reported that sport was an integral part of their family culture. This involved other family members being former professional athletes and/or older siblings actively taking part in sports.

Early years experiences typically involved regularly taking part in a range of fun and challenging activities and attending/watching sporting events. Participants also reported playing sporting activities in the back garden or the local park. These activities were largely organised and supported by the parents. All participants believed that these family activities had positively facilitated their motor skill development as well as contributed to their motivation and positive attitude. In addition, participants suggested that the challenging and competitive activities had contributed to the early development of their aptitude. Importantly, playing for a team from an early age was reported as having an important impact on their development. All reported participating in a range of activities from an early age and this multisport experience was also perceived to have contributed to their successful development within their main sport.

All but three participants reported experiencing early success which was perceived to have contributed to their confidence levels. Notably, this early success was influenced by positive feedback provided by parents or being identified as a talent from an early age.

### Challenges

Early challenges appeared to be highly idiosyncratic, with young performers' interpretations on what constitute a challenge varying greatly. Sport challenges constituted the largest proportion of the experiences reported as having an important impact on their development. As per Table 4, an early memorable challenge consistent amongst players in both sports was the intense period of academy trials. Being selected to play for an older age group, was another early sport challenge that the participants commonly referred to within their interviews followed by underperformance*.* In essence, participants described the difficulties experienced when they had to compete against older players and the emotional upheavals resulting from poor performances. Importantly, playing a year up reportedly carried secondary challenges, such as those of physical nature. Besides this, however, the occurrence of *physical* challenge was also described as being determined by other factors, including sport-specific demands and individual differences. Indeed, some participants encountered physical challenges due to them being less physically developed than their counterparts. This was particularly challenging for rugby participants where the nature of the sport, which requires high levels of strength and physicality, can add-up to a potential existing handicap resulting from a less developed physical outlook “You know I was small and, obviously, other players would outplay me in most game scenarios. Rugby requires physicality. I mean it is a full-contact sport.” (R1)

Within sport challenges, having to adapt to games changes was particularly challenging for some participants whilst rejection was also identified as an early hurdle. Whilst injuries can be an inherent part of sports participation, players indicated that being forced to stay out of sports due to an *injury* could be immensely challenging, especially at an early age. Finally, less frequent among challenges of a sporting nature, and perhaps indicative of the highly idiosyncratic incidence of early experience, were challenges concerning a late start in their main sport and decisions about choosing between academies.

In addition to the sport related challenges described above, a large proportion of participants indicated some social/environmental challenges that seemed to have had an impact on their development. Interestingly, however, all those challenges appeared to be closely linked to and/or a result of sport involvement. For example, settling into a new team at an early age seemed to be a stressful experience for many participants. In addition, even though siblings and friends can act as a strong support network for young developing athletes, they may also be a significant source of stress. This was discussed by participants who identified perceived criticism from significant others as a profoundly difficult experience that could initially affect their emotional states. Along the same lines, some participants described the levels of *pressure* experienced when significant others verbalised their unrealistic expectations of them. Finally, the challenge of having to leave friends behind was preceded by a decision to choose between academies and/or change a sporting environment.

Whilst less pervasive throughout the data, perhaps due to the unforeseen and serendipitous nature of them, some participants referred to family challenges as having an impact on their development. This predominantly involved incidents of bereavement with unpredictable *illness* far less common. Even though reported, however, the rate of incidence was far less than suggested by some literature [e.g., (46)] and more in keeping with other concurrent work [e.g., (10)].

Devoting time to sports can often bring some additional challenges for the young performers. For instance, the challenge of *balancing commitments* was recognised by four participants. Less evident throughout the data were logistical issues concerning *travel commitments.* Finally, another challenge that emerged as a result of sport involvement was *drops in school performance,* although this was only reported by one participant.

## Discussion

Coping skills have been shown to play an influential role in helping young individuals to realise potential in various performance domains such as sport, business and dance (47–49). The purpose of this study was to deepen understanding about the extent to which young players arrive with skills or as a blank canvas when entering an academy. The design of this study allowed participants to explore their reflections of their experiences and skills developed and utilised before entry.

Notably, the findings of this study contribute to a deeper understanding of the growing area of TD research (4, 14, 16, 17) by showing that young performers arrive at the academy pathway with a fledging set of coping skills. Interestingly, there appears to be a stark similarity between the nature of coping skills reported in the present paper and those previously identified in the TD literature. More specifically, mental skills found in this study (e.g., motivation, self-efficacy, focus and distraction control) overlap with those reported in prior work (4, 7, 8, 14, 50, 51). Unsurprisingly, motivation and self-efficacy seemed to be particularly important in the early years, perhaps due to them being key drivers of commitment and involvement. In addition, although the importance of diverse and coherent social support is well-established in TD literature [e.g., (15, 52)], our results indicated parents as the primary “providers” of this support in pre-academy years [cf. (53)]. Moreover, the key role of learning skills through the use of metacognitive strategies (e.g., reflection, evaluation) has been extensively discussed in prior work (7, 9, 54). Taken collectively, such findings further solidify previous suggestions about the importance of psychological skills, social support and learning skills in facilitating the development of talent. However, the level and confidence in ability to use the skills may not be necessarily identical to that of more experienced athletes reported in previous studies [e.g., (4, 14)].

Further, our findings provide a novel insight into the genesis and early development of these coping skills before entry, a matter, until now, which has remained largely unconsidered. More specifically, the results suggest that children, as a result of *general* experiences and upbringing, acquired an aptitude which then became synthesised into a more focused skill as a result of *specific* challenges. In terms of the nature of these general experiences, these findings support evidence found in the physical literacy literature. Indeed, the benefits of early motor skill development and physical competence have long been linked to heightened confidence (55) and increased motivation to maintain physical activity throughout life (56, 57). Moreover, early success has been associated with confidence and motivation in sport (58). Finally, the early experience of working in teams can have many benefits such as acquiring social skills (59), positive self-perceptions, enjoyment, and persistence (60).

Within the spectrum of general experience, parental involvement and associated behaviours seemed to play a key role in the development of this aptitude, supporting previous findings in the area. Beyond the influential role parents can play in socialising children into sport (61), they can also influence their child's psychological functioning and cognition in sport [e.g., (62–64)]. Recently, Teques et al. (65) found that parental support through the use of reinforcement, modelling and encouragement was associated with higher levels of self-efficacy, social efficacy, intrinsic motivation, and self-regulation among young performers in a range of team and individual sports. Indeed, parents are the main source of influence during this time (66), whilst young performers develop their perceptions of physical competence and self-confidence that can aid both their long-term development through sport and their chances of maximising their potential (67, 68). Ideas of strength-based parenting are also clear from our data [cf. (69)] whilst the lack of intrusion style also match earlier research [e.g., (10)].

Moreover, these findings support previous work in adversity-related growth literature [e.g., (4, 70)]. Unlike TD environments. Importantly, however, challenges experienced before entry appeared to offer an opportunity for young athletes to turn an aptitude acquired from early general experiences and upbringing into a more focused set of skills to solve particular issues. This could shed light on the ongoing debate over the mechanisms that underpin growth following adversity (e.g., 73). More specifically, our findings suggest that previous suggestions regarding the role of challenges should not be seen as opposing poles of the same stick but rather as complementary views that address the same matter. As mentioned, some mental skills and social support were present before the occurrence of challenge [cf. (25)]. Notably, when faced with a challenge, participants relied on previous personal and/or others' experiences to inform their response. Importantly, the process by which meaningful lessons were drawn through the use of reflective practice indicates that, while challenges can set the preconditions for subsequent learning and development to occur (70), it is also the learnt skills that young athletes deploy to deal with those challenges that seems crucial (10, 25).

Whilst there seemed to be key similarities in the types of early general experiences that enabled young athletes to develop an aptitude, the nature of challenge that underpinned the subsequent synthesis of more focused skills were highly idiosyncratic. In line with prior work that focused on the experience of athletes whilst on the TD pathway (4, 14, 70, 74), the nature of the challenges recalled in this study were complex, including both sporting and non- sporting issues.

Regarding the timing of the challenges, these findings extend current understanding of perceived trauma occurring at the early stages of sport participation which contrast previous assumptions reported by Savage et al. (4) positing that perceived traumas occur no earlier than 7 years after commencing a sport. To clarify, Savage et al. (4) contended that perceived and memorable traumas can be seen as those experienced in the initial phases of investment (75), perhaps because the impact of challenge may be greater at this stage due to the high levels of time and commitment invested by young athletes. However, our findings indicate that young athletes (at least these who were identified as of greater potential) not only face challenges in sampling years, but that those challenges seem to play a pivotal role in the subsequent development and refinement of their existing coping skills.

Reflecting this, there are several messages from the ways in which skills are acquired and implications for TD environments. The results from this study are in contrast to the sole and formal construct of post-traumatic growth or as suggested by Collins et al. (10) teach-test-tweak-repeat. Of course, that is not to say that this does not happen, nor that this is not a useful strategy for performers once in the academy. Instead, our findings posit that upbringing and appropriate general experiences have led to an aptitude or tendency (cf. Proactive coping – 76). That aptitude is then utilised to solve particular issues, such as different developmental challenges. The impact of early familial and general sport experiences is much more akin to physical literacy because the armoury of skills is, unsurprisingly, not taught in a formal sense.

As such, if children are arriving at academies with an established aptitude and an early version of skills, it is important that early on practitioners assess athletes’ skill provision, or toolbox, and use this as a starting point for development. Additionally, information regarding the types of experiences and challenges young athletes face prior to embarking on the TD process could facilitate the deployment of appropriate experiences. Indeed, akin to recent calls for strategies that would optimise TD structures (14), this information should provide the platform for TD stakeholders to individualise young athletes' pathway through preparation processes (assessing, teaching), reflection processes (tweaking) and, ultimately, periodisation of developmentally appropriate challenges in the early years of the talent pathway. Of course, it is important to note that there are more implications regarding parenting, early sporting development and pre-academy pathway structures, however this sits outside the scope of this paper and would warrant further investigation.

Supporting the call for longitudinal and prospective research in TD (73, 11), it would be beneficial to our understanding to follow up with these players to identify how they then continue to develop once they are in a much more structured and skills-focused level of the academy. Essentially, it may be important to closely investigate the evolving and dynamic nature of negotiating difficult challenges as young athletes' transition further on the talent pathway. Research on this topic could also compare the skills of talented athletes in different professional sport contexts. For example, players enter the academy system at a much later age in rugby, which may suggest that young rugby players arrive with more established set of skills at the academy as a result of broader experiences and challenges encountered.

Of course, this study was not without limitations. For example, due to the retrospective nature of data collection, there is a risk of recall accuracy (77, 2005). Although careful steps were taken to proactively address some of these limitations (e.g., recruiting participants who have recently arrived at the academy system), participants recalling events that occurred years or months ago can have resulted in lower accuracy of reports. Moreover, given the personal nature of reporting highly experiential accounts, there is a risk of impression management. That is, participants may have provided overly positive and growth-stimulating accounts of their pre-academy experiences, including challenges encountered and skills acquired/deployed, compared to their actual experiences. Furthermore, no steps were taken to triangulate the skills and experiences of the participants with questionnaires and/or with views from significant others such as parents and coaches. This would have potentially provided a richer picture into the skills that those young athletes arrived at the academy as well as the experiences through which those skills were acquired. Finally, it should be stressed that all participants were “highfliers” within the academy. It may be that, were we to have examined their less successful colleagues, a less positive picture would have emerged.

Notwithstanding the above shortcomings, as approaches to optimise trustworthiness (cf. Methodology section), methodological coherence was aided by framing our questions, purposeful selection of participants, methods, analyses, and interpretation in a pragmatic research philosophy (29). In accordance with our pragmatic philosophy (29), we believe the findings of this study provide contribution to practice-oriented theory and consultancy.

In conclusion, this study provided evidence that young athletes arrive at the academy with an early version of coping skills. Whilst the influential role of coping skills in facilitating learning and development is well-reported in prior TD work (1, 14, 16, 78), this study also investigated how young athletes learn, develop and deploy coping skills in much less structured environments such as the pre-academy life. Finally, talent pathway practitioners may significantly benefit from systematically assessing the young athletes' skills and pre-entry experiences upon arrival. This will enable these practitioners, supported by all stakeholders, to subsequently develop and deploy tailored programmes to support young athletes at the beginning of and along the pathway.

## Data Availability

The raw data supporting the conclusions of this article will be made available by the authors, without undue reservation.
